# Dual-biased metal oxide electrolyte-gated thin-film transistors for enhanced protonation in complex biofluids

**DOI:** 10.1038/s41598-024-80005-0

**Published:** 2024-12-28

**Authors:** Chuljin Hwang, Yoonseok Song, Seokhyeon Baek, Jun-Gyu Choi, Sungjun Park

**Affiliations:** 1 Department of Electrical and Computer Engineering, Ajou University, Suwon, 16499 Republic of Korea; 2 Department of Intelligence Semiconductor Engineering, Ajou University, Suwon, 16499 Republic of Korea

**Keywords:** pH sensor, Dual-biased electrolyte-gated thin-film transistors, Oxide semiconductor, Back-gate effect, Biofluids, Engineering, Materials science

## Abstract

pH sensing technology is pivotal for monitoring aquatic ecosystems and diagnosing human health conditions. Indium–gallium–zinc oxide electrolyte-gated thin-film transistors (IGZO EGTFTs) are highly regarded as ion-sensing devices due to the pH-dependent surface chemistry of their sensing membranes. However, applying EGTFT-based pH sensors in complex biofluids containing diverse charged species poses challenges due to ion interference and inherently low sensitivity constrained by the Nernst limit. Here, we propose a dual-biased (DB) EGTFT pH sensing platform, acquiring back-gate-assisted sensitivity enhancement and recyclable redox-coupled protonation at the semiconductor-biofluid interface. A solution-processed amorphous IGZO film, used as the proton-sensitive membrane, ensures scalable uniformity across a 6-inch wafer. These devices demonstrate exceptional pH resistivity over several hours when submerged in solutions with pH levels of 4 and 8. In-depth electrochemical investigations reveal that back-gate bias significantly enhances sensitivity beyond the Nernst limit, reaching 85 mV/pH. This improvement is due to additional charge accumulation in the channel, which expands the sensing window. As a proof of concept, we observe consistent variations in threshold voltage during repeated pH cycles, not only in standard solutions but also in physiological electrolytes such as phosphate-buffered saline (PBS) and artificial urine, confirming the potential for reliable operation in complex biological environments.

**Supplementary Information:**

The online version contains supplementary material available at 10.1038/s41598-024-80005-0.

## Introduction

Rising interest in environmental and public health has spurred advancements in pH sensing technology, enabling monitoring of aquatic ecosystems, biological activity, healthcare products, agriculture, and life science^1–5^. Notably, the pH levels in bodily secretions like urine, saliva, and sweat provide vital insights into human health, aiding in point-of-care diagnostics for various diseases^6,7^. Since the development of colorimetric paper-based pH indicators, which suffer from quantitative inaccuracy, commercial pH meters with proton-sensitive glass electrode membranes have provided wide scanning ranges, improved accuracy, and chemical robustness. However, their limited portability, fragility, low sensitivity, and maintenance costs for storage has led to an exploration of transistor-based pH sensors^8,9^. Ion-sensitive field-effect transistors (ISFETs) represent the traditional device architecture in pH sensing devices, employing a distinct sensing membrane between the electrolyte and the active layer^10,11^. In contrast, in organic electrochemical transistors (OECTs) and electrolyte-gated thin-film transistors (EGTFTs), the active layer itself serves as sensing membrane, depending on whether the active materials are organic or inorganic^12–14^.

Among these, EGTFTs have garnered significant attention as pH sensing devices^15,16^. This configuration leverages the high capacitance and signal amplification from the electric double layer (EDL) formed at the electrolyte-semiconductor interface, allowing for rapid response and low-voltage operation below 0.5 V without causing water electrolysis^17,18^. Metal oxide (MO_x_) semiconductors have emerged as promising active materials in EGTFTs due to their streamlined fabrication process via affordable solution-processing, non-toxicity, superior chemical robustness, and quick responsiveness^19–22^. Beyond their material robustness, MO_x_ semiconductors exhibit pH-sensitive interfaces themselves, as explained by the site-binding theory^23^. The hydroxide groups on the surface of MO_x_ semiconductors undergo protonation or deprotonation depending on the pH conditions of the electrolyte. The pH sensitivity (*S*) can be described as *S* = Δ*V*_TH_/ΔpH, where Δ*V*_TH_ is the threshold voltage shift identical to the surface potential difference (Δ*φ*) in EGTFTs^24^. Ideally, the theoretical upper limit of *S*, known as the Nernst limit, is 59.16 mV/pH at 25 °C, constrained by intrinsic thermodynamic, kinetic factors and/or surface site densities^25^.

Since pH sensitivity is significantly dependent on effective surface area, structural engineering approaches, including nanoparticle decoration and/or low-dimensional active layers, have been explored^26–28^. The substantially enlarged surface area provides increased interaction sites for proton in electrolytes, enhancing sensitivity. Another intuitive approach is to expand Δ*V*_TH_ by enhancing surface reactivity, based on surface functionalization of sensing membranes^29^. Amine groups (–NH_2_) on MO_x_ semiconductor, tailored by self-assembled monolayers, exhibit a strong propensity for protonation at low pH levels, converting to positive–NH_3_^+^ groups. This leads to electron accumulation in the channel, and consequently more negative shift in *V*_TH_. Additionally, the electrical amplification by connection of independent transistor with sophisticated fabrication process has also proven effectiveness to enlarge Δ*V*_TH_, resulting in sensitivity enhancement^30–34^.

However, achieving reliable and highly sensitive pH sensors in complex biofluids containing diverse ion species remains a significant challenge. Incorporation of nanoparticles in sensing membranes often suffers from operational reliability issues in concentrated solutions, attributed to undesired interactions with other ion species. Additionally, devices with surface-functionalized membranes are vulnerable to interference from these charged components, leading to gradual *V*_TH_ drift under repeated pH cycling^25,35^. Furthermore, selecting the appropriate sensing materials connected to the transistor for electrical amplification is critical for prolonged submersion in biofluids, as improper choices can hinder sustainable signal reading. Therefore, innovative strategies should be considered to enable practical applications of pH sensors in complex biological environments with high sensitivity beyond the Nernst limits.

In this study, we present a dual-biased (DB) EGTFT pH sensing platform with a unified structure, revealing high sensitivity and operational stability in physiological electrolytes. Solution-processed IGZO semiconducting layer served as both active layer and sensing membrane, ensuring wafer-scale uniformity as well as chemical robustness in standard pH solutions. Following investigations into how dual biases affect protonation of the IGZO film, in-depth analyses using electrochemical impedance spectroscopy (EIS) elaborate on the charge accumulation mechanism, leveraged by pH and back-gate voltage (*V*_BG_), respectively. Without any surface functionalization of the IGZO film, we achieved enhanced sensitivity beyond the Nernst limit under alternating pH levels between 4 and 8 at constant *V*_BG_. As a proof-of-concept, phosphate buffered saline (PBS) and urine with varying pH levels were used as electrolytes, corroborating the capability of these devices for pH monitoring in biological conditions. Our fundamental approach to understanding the pH sensing mechanism in relation to device architecture is anticipated to open new avenues for environmental monitoring systems and point-of-care biomedical applications.

## Experimental section

### Preparation of IGZO solution

Indium nitrate hydrate (In(NO_3_)_3_∙xH_2_O), gallium nitrate hydrate (Ga(NO_3_)_3_∙xH_2_O), zinc acetate dihydrate (Zn(CH_3_COO)_2_∙2H_2_O), and 2-methoxyethanol were purchased from Sigma–Aldrich and used without further purification. All the measurements were conducted in a dark chamber, and the temperature was pinned at 25 ± 1 °C. The IGZO solution was prepared at a fixed concentration (0.1 M), and the molar ratio of In: Ga: Zn was set to 0.1:0.15:0.0275 in 2-methoxyethanol. To ensure the formation of a homogeneous oxide compound, the solution was stirred at 60 °C for 4 h.

### Fabrication of IGZO EGTFTs

Thermally oxidized SiO_2_ layers of thickness 300 nm on highly doped Si (p^++^) wafers were prepared as substrates. The patterned Au electrodes were deposited on as-cleaned substrates by conventional photolithography and thermal evaporation processes with a channel width and length of 200 and 10 μm, respectively. The as-prepared IGZO solution was filtered using a syringe filter and spin-coated onto the Au-patterned substrate. The IGZO films were then thermally annealed on a hot plate at 400 °C for 1.5 h. Subsequently, the IGZO films were isolated by conventional photolithographic processes and passivated by the patterned SU-8 layer.

### Preparation of pH solutions

Prior to electrical characterizations of the EGTFT-based pH sensors, we prepared a PDMS well to isolate an electrolyte. The pH levels were set using standard pH solutions (4–8 of pH levels, Duksan). Artificial urine and PBS solutions were used for physiological electrolytes. The test solutions including standard pH solution, PBS solution, and artificial urine were placed in a water bath at 25 °C and measurements taken only at this temperature. We leveraged the pH levels of physiological electrolytes to modulate their acidity by carefully titrating sulfuric acid (ACS reagent, 95.0–98.0%, Sigma-Aldrich) and sodium hydroxide (ACS reagent, ≥97.0%, Sigma-Aldrich), while simultaneously monitoring with commercial pH meters.

### Electrical characterization

Electrical characterization was performed using a Keithley 2602B dual channel sourcemeter (Keithley, Cleveland, USA) under ambient conditions in a dark chamber. All the potentials were biased with respect to the Ag/AgCl reference electrode (RE) (CHI111, CH Instruments, USA). The EIS measurements were conducted to analyze the impedance changes between the IGZO surface and pH solution. The experimental data were collected using a CS350 electrochemical workstation (CorrTest, Wuhan, China). During EIS measurements, the voltage of the RE ranged between − 0.5 V and 0.5 V with a scan rate of 10 mV/s. The frequency range used in the study was 0.01–10 Hz. These parameters enabled a comprehensive impedance analysis of the IGZO surface and its interaction with the pH of the solution.

## Results and discussion

### pH sensing performance of IGZO EGTFTs

**Fig. 1:**
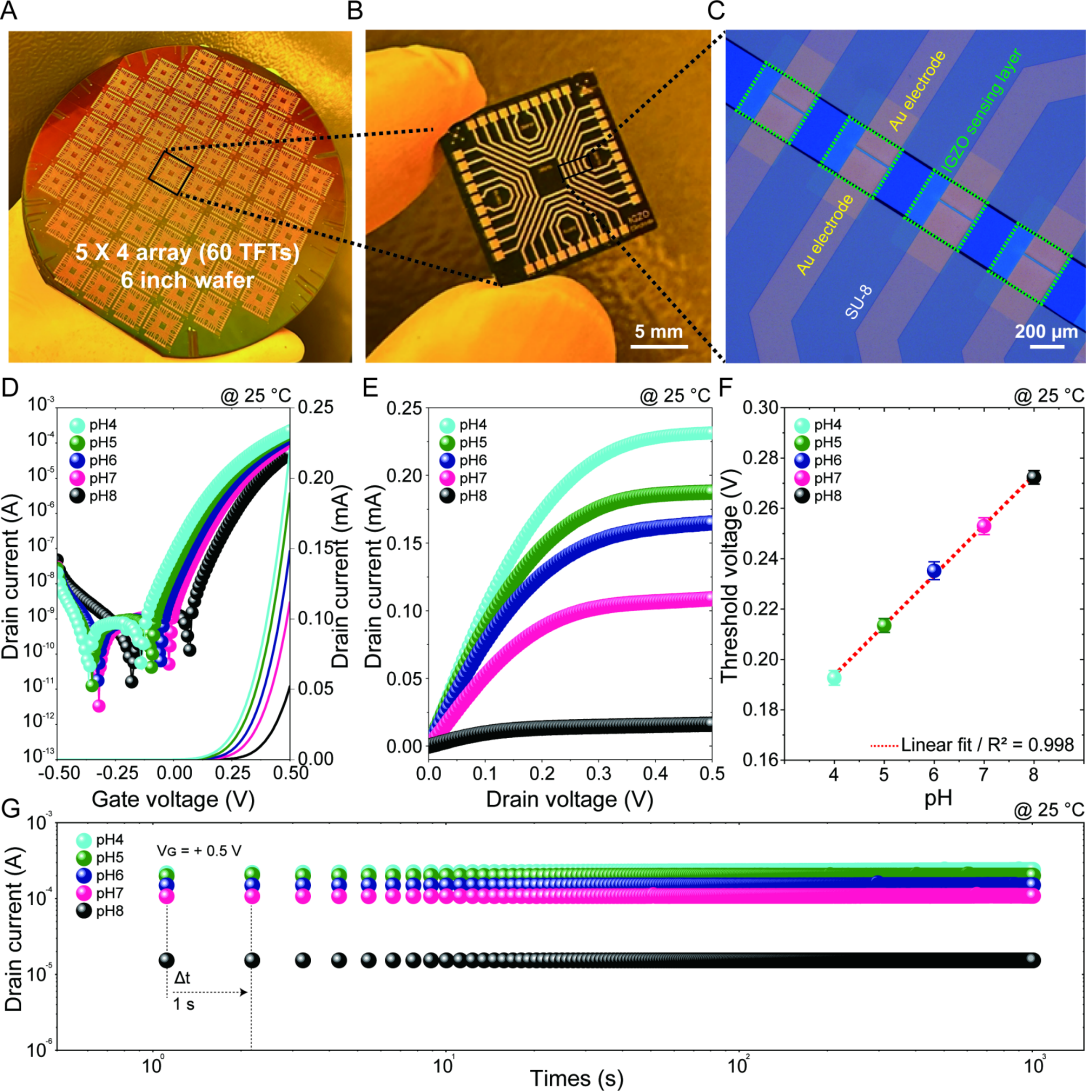
Wafer-scale fabrication and electrical properties of pH sensor based on indium-gallium-zinc-oxide electrolyte-gated thin-film transistors (IGZO EGTFTs). (**A**) Photograph image of IGZO EGTFTs on a 6-inch Si/SiO_2_ (300 nm) wafer substrate (in total, 60 cells were fabricated). (**B**) Schematic illustration of a single chip (1.5 × 1.5 cm^2^) consisting of 20 EGTFTs and respective interconnected pads. (**C**) Microscope image of individual IGZO EGTFTs with source/drain (*S*/*D*) Au electrodes (yellow character), SU-8 passivation (white character), and IGZO sensing layer (green character and dot). (**D**) Transfer and (**E**) output characteristics (*V*_TG_ = 0.5 V) by leveraged pH levels from 4 to 8. (**F**) The plots of threshold voltage (*V*_TH_) of IGZO EGTFT (*V*_D_ = 0.5 V) against pH levels. The dashed red line indicates a linear fitting from the experimental data. The error bars were calculated from three parallel experiments. (**G**) Dynamics of drain current (*I*_D_) under different pH with the time intervals of 1 s, where the *V*_G_ and *V*_D_ were fixed at 0.5 V.

First, we verified the spatial uniformity of the IGZO EGTFTs fabricated on a 6-inch wafer. A single wafer contains 60 cells (Fig. 1A), and a single cell includes 20 EGTFTs within an area of 1.5 × 1.5 cm^2^ (Fig. 1B), which corresponds to a total of 1200 EGTFTs on a single 6-in wafer. Figure 1C shows a magnified optical microscopic image of the EGTFTs and the IGZO channel area atop the source and drain (S/D) electrodes. The IGZO layer was selectively exposed within an active area of 2000 μm^2^ through the conventional photolithography process and simultaneously passivated by SU-8 to circumvent short-circuits arising from the direct interaction between the liquid electrolyte and S/D electrodes.

To evaluate the electrical characteristics of IGZO EGTFT, in which only the top-gate voltage (*V*_TG_) was applied, we dropped an electrolyte droplet with a fixed volume of 20 µL onto the channel region using a micropipette in the prepared polydimethylsiloxane (PDMS) well, and then submerged Ag/AgCl RE into the electrolyte for gate biasing. Figure S1A shows the representative transfer characteristics of the IGZO EGTFT, where the pH was set at 7, showing exceptional on/off current ratios (*I*_on_/*I*_off_) exceeding 10^8^. Meanwhile, the output characteristics exhibited a linear trend below the pinch-off drain voltage (*V*_D_), thus indicating an ohmic contact between the IGZO layer and the S/D electrodes (Figure S1B). Moreover, the increased drain current (*I*_D_) by increasing gate voltage (*V*_G_) revealed an n-type transistor behavior with the accumulation mode. Figure S2 displays the counterclockwise hysteretic curves between the forward and backward voltage sweeps. A negligible hysteresis loop suggests that the electrolyte-interfacial and bulk traps of IGZO layer could remain in an equilibrium state during the alternation of the electric field within the sweep rate of the *V*_G_. Consistently, Figure S3A shows the sequentially measured transfer curves over 60 cells in a solution with pH = 7, where the gate bias was applied through the RE without the back gating effect. For the statistical investigations, we extracted key parameters such as the maximum transconductance (*g*_m_), subthreshold swing (*SS*), and threshold voltage (*V*_TH_) from the transfer, whose mean values were determined to be 0.28 mS, 83.0 mV/dec, and 0.25 V, respectively (Figures S3B − D), highlighting the exceptional reliability and uniformity of solution-deposited IGZO over a 6-inch wafer scale.

To empirically verify the capability of the IGZO EGTFTs as pH sensors, we varied the pH of the electrolyte and measured the electrical characteristics. Note that the sweep range of the *V*_TG_ was carefully chosen to avoid water electrolysis, which typically necessitates a minimum potential difference of 1.23 V^12^. Figure 1D and E show the transfer and output curves of the IGZO EGTFTs with different pH levels from 4 to 8 at standard temperature (25 °C). The lower pH electrolyte led to the negative shift of transfer curves with enhanced drain current. Typically, the pH sensitivity can be determined using the relative surface potential difference with respect to unit change in pH, expressed using the following equation:^31,36^

1 $$\:\frac{d{\phi\:}_{0}}{dpH}=-2.303\alpha\:\frac{kT}{q}$$

where $\:{\phi\:}_{0}$ represents the surface potential, *α* is a dimensionless sensitivity parameter that varies between zero and one, *k* is the Boltzmann’s constant, *T* is the absolute temperature, and *q* is the elementary charge. Meanwhile, the Δ*V*_TH_ can be expressed as the inverse value of surface potential difference, given that the *V*_TH_ is equal to the flat band voltage at the semiconductor-electrolyte interface^37^. Figure 1F shows a plot of the *V*_TH_ of the IGZO EGTFTs as a function of pH, and it indicates a strong linear relationship (*R*^2^ = 0.998) with a slope of 20 mV/pH, which is the sensitivity.

Another crucial aspect of pH sensors is their operational stability. Figure 1G shows the dynamic plots of *I*_on_ at different pH levels, where *V*_TG_ = 0.5 V and *V*_D_ = 0.5 V, indicating negligible changes after recording for 10^3^ s. This consistency confirms that the sol-gel-derived IGZO is chemically robust, thereby leading to prolonged reliability of EGTFTs over 10^3^ s. This period aligns with the minimum settle-down time required to complete the process of sensing biological analytes in potential applications^38^.

### Back-gate effects on IGZO EGTFTs

**Fig. 2:**
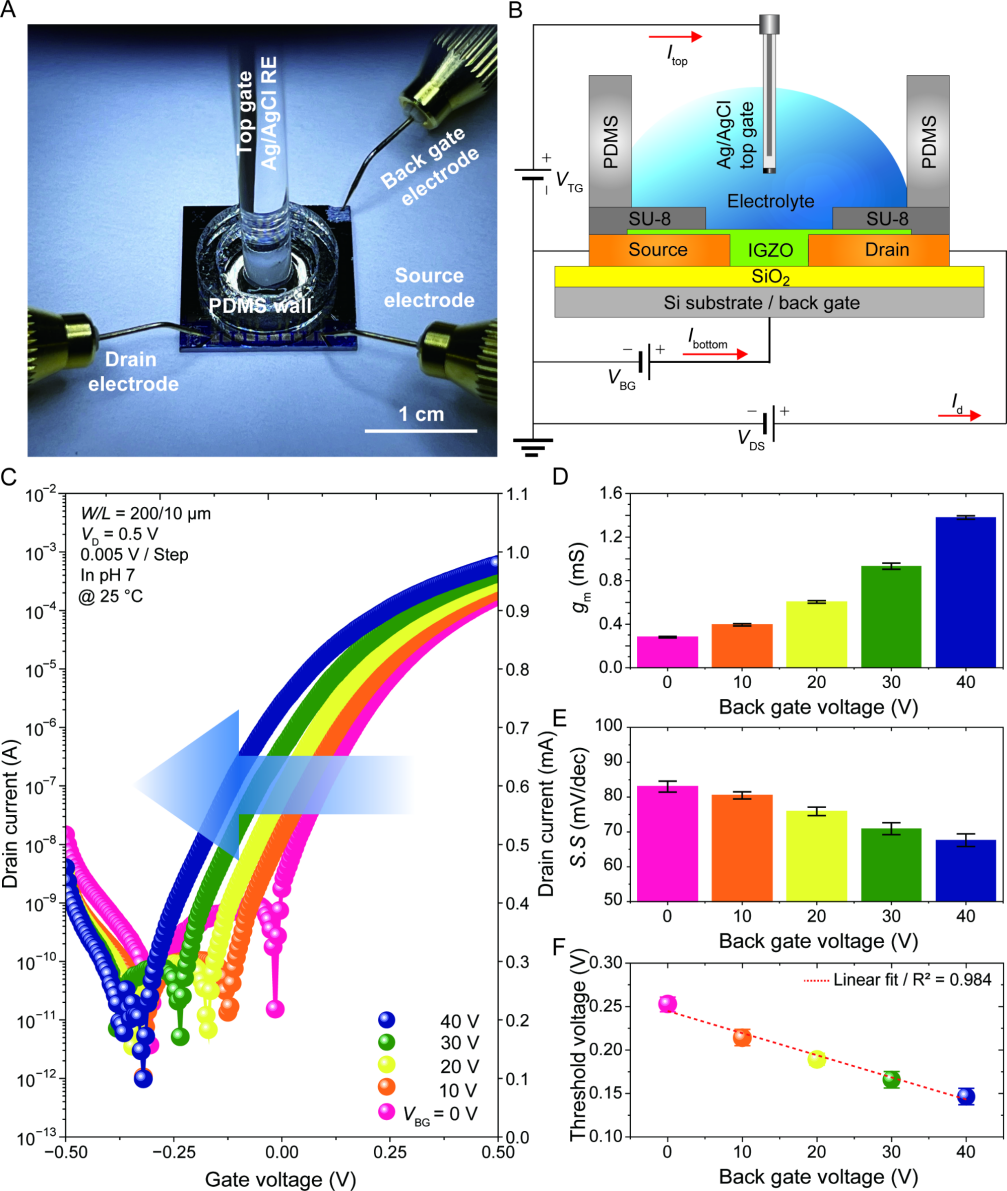
Characterization of electrical properties of pH sensors. (**A**) Photograph showing the moment for electrical measurement of DB EGTFTs. (**B**) Schematic configuration showing the cross-sectional structure and electrical connections of the DB EGTFTs. (**C**) Transfer characteristics of DB EGTFTs at *V*_D_ = 0.5 V with different back gate voltage (*V*_BG_), where pH is pinned at 7. Semiconductor parameters extraction of (**D**) *g*_m_ and (**E**) *SS* by leveraging *V*_BG_ from 0 V to 40 V; error bars denote a standard deviation for a total of 50 devices. (**F**) The *V*_TH_ plots of DB EGTFTs (*V*_D_ = 0.5 V) against *V*_BG_. The dashed red line indicates a linear fitting from the experimental data. The error bars were calculated from three parallel experiments.

Although the sol-gel-derived IGZO exhibited outstanding chemical robustness with operational stability, the pH sensitivity of a single-gate EGTFT is highly insufficient for surpassing the Nernst limit. Therefore, we designed an architecture with dual biases to achieve significantly enhanced sensitivity. Figure 2A presents a photographic image at the moment of electrical measurement of the DB EGTFT, and Fig. 2B shows a schematic structure of the proposed DB EGTFT. Our devices employ two distinct gate electrodes: (1) an Ag/AgCl RE as the top gate, immersed in an electrolyte to drive the EDL, and (2) a highly doped silicon as the back-gate. Prior to investigating DB EGTFTs, we tested the *V*_BG_ effects of IGZO TFTs to demonstrate the n-type characteristics of IGZO without electrolyte effects. Figures S4A−C showcase the representative device structure, transfer, and output curves of the IGZO thin-film transistors (TFTs), respectively, revealing a typical n-type behavior with an accumulation mode.

Subsequently, we evaluated the effects of back-gate bias by varying *V*_BG_ from 0 to 40 V, and sweeping the *V*_TG_ from − 0.5 to 0.5 V at *V*_D_ = 0.5 V. Figure 2C plots the transfer characteristics of the IGZO EGTFTs with different *V*_BG_ at pH = 7. Note that conventional dual-biased EGTFT pH sensors exploited an extended gate architecture that separately isolates the transducer and sensor units, fixing *V*_TG_ through a reference electrode and sweeping *V*_BG_ to significantly expand the Δ*V*_TH_^30–34^. In contrast, the IGZO serves a dual role as both the sensing and active layer. This novel architecture not only ensures wafer-scale uniformity and reliability but also simplifies the integration process, making it promising for point-of-care diagnostics of biofluids. Despite a fixed pH, an increase in *V*_BG_ led to a gradual shift of the transfer curve toward a negative direction. The electric field driven by *V*_BG_ results in a higher charge accumulation in the IGZO channel, indicative of an electron doping effect with negative *V*_TH_ shift. We extracted various figure-of-merit parameters such as *g*_m_, *SS*, and *V*_TH_ from the transfer curves with different *V*_BG_, achieving the highest *g*_m_ (1.38 mS) and lowest *SS* (67.63 mV/dec) at *V*_BG_ = 40 V, as shown in Fig. 2D and E. Meanwhile, Fig. 2F shows a well-fitted linear relationship between the *V*_TH_ and *V*_BG_ with *R*^2^ value of 0.984. For a dual-gate transistor, the total charge (*Q*_tot_) accumulated in the channel can be simultaneously affected by both gate biases as:

2 $$\Delta Q_{tot}=C_{B} \Delta V_{BG} + C_{T} \Delta V_{TG}$$

where *C*_B_ and *C*_T_ represent the SiO_2_ and electrolyte interfacial capacitances, respectively. Assuming a constant pH and fully depleted channel at *V*_TG_ = *V*_TH_, which implies neither change in *C*_T_ nor more charge accumulation, the net charge difference becomes zero^33^. Therefore, the *V*_TH_ value is given by,

3 $$\Delta V_{TH} = - (CB/CT) \Delta V_{BG}$$

This equation elaborates on the linear relationship between *V*_TH_ and *V*_BG_, where the slope of the linear fit was 2.67 mV/V. Considering that the pH sensitivity is governed by Δ*V*_TH_, a strong electric field from *V*_BG_ is regarded as a promising driving factor to enhance the sensitivity beyond the Nernst limit.

### Electrochemical analysis of IGZO–electrolyte interface

**Fig. 3:**
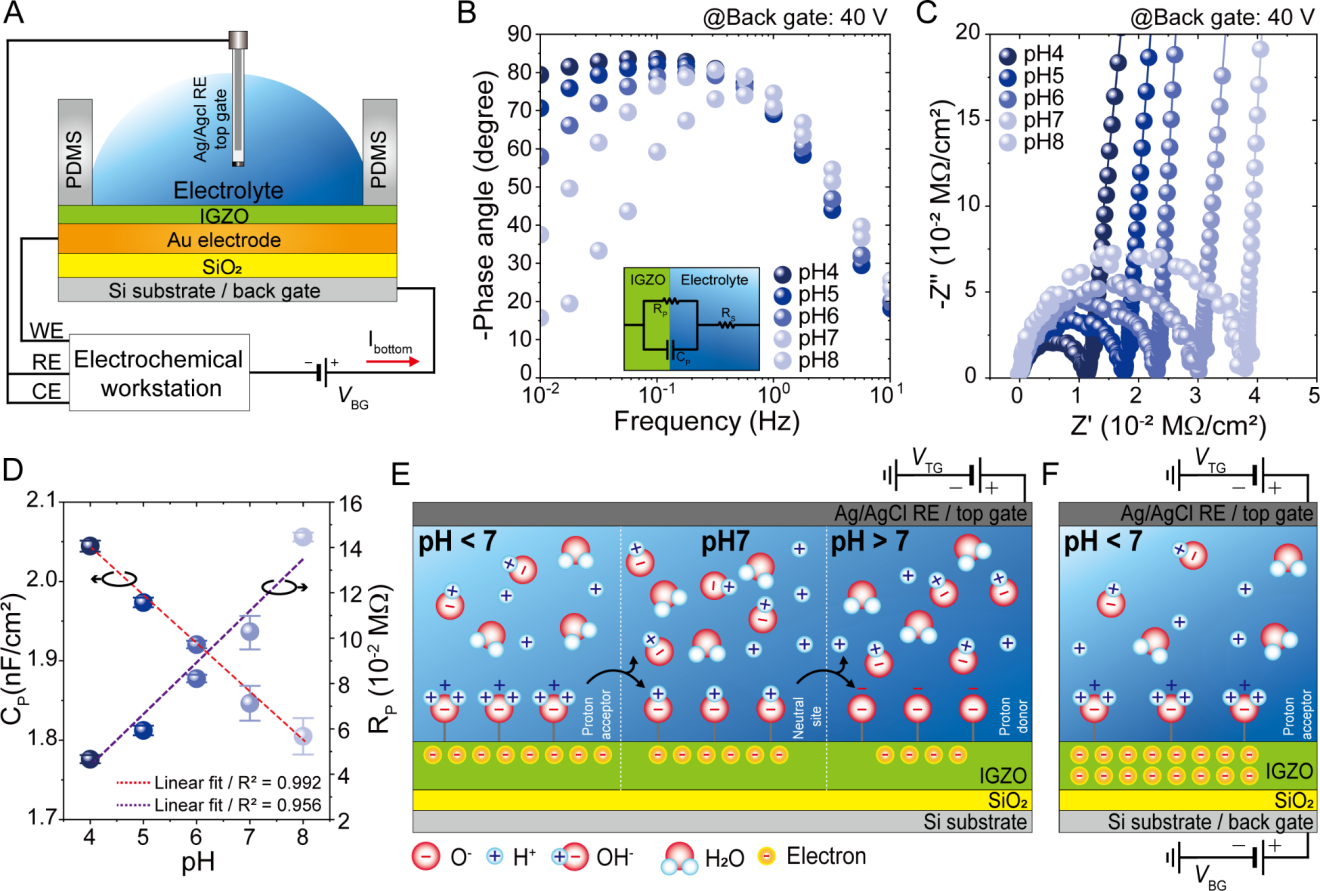
Electrochemical impedance spectroscopy (EIS) analysis using solutions with various pH levels. (**A**) Schematic of the experimental setup of EIS and electrical connections of the working reference and counter electrodes. The Ag/AgCl RE was utilized as the RE to inhibit the creation of an EDL at the interface between the Ag/AgCl RE and the electrolyte. (**B**) Bode phase showing the effect of pH on the electrochemical and semiconducting properties of the interface between IGZO and electrolyte. In the inset, an equivalent electrochemical circuit is depicted. (**C**) Nyquist plot displaying the real (*Ζ*′) and imaginary (*Ζ*′′) components of impedance for the IGZO-electrolyte-Ag/AgCl RE configuration. The points indicate the experimental data, while the solid lines of matching colors represent the fitting outcomes. (**D**) Extracting resistance and capacitance parameters with different pH solutions. Schematic of the pH sensing mechanism at the IGZO-electrolyte interface according to pH solution with (**E**) top gate biasing and (**F**) top and back-gate biasing.

To understand the operational mechanisms of achieving the enhanced sensitivity, we investigated the pH-dependent EDL characteristics using the EIS measurement. All measurements were performed at standard temperature (25 °C) in a dark chamber to circumvent any photon-induced and/or electrical interference. Figure 3A shows a schematic of the device architecture used for the EIS measurements. The Au layer underlying IGZO served as the working electrode (WE), and the electrolyte was connected to both the counter electrode (CE) and RE^39^. Figure 3B and C present two distinct standard types of EIS plots, Bode and Nyquist plots, where the pH of electrolyte is varied from 4 to 8, under dual-gate operation conditions (*V*_BG_ = 40 V). In the Randle equivalent circuits, which are depicted in the inset of Fig. 3B, the *R*_S_, *R*_p_ and *C*_p_ denote the electrolyte resistance, charge transfer resistance at the interface, and EDL capacitance, respectively. Interestingly, the Bode phase angle plots presented that the change in pH led to distinct behaviors of phase angle at low frequency ranges. Because the EDL originates from the ionic interactions and movement at the interfaces, the *C*_P_ is significantly more susceptible in the low frequency range than in the high frequency range. Therefore, the declining phase angle toward zero in low frequency range indicates a low capacitive response of EDL, which corresponds to the increase in *R*_P_ by increasing the pH level. Consistently, a growing radius of semicircle in the Nyquist plots demonstrated the rise in *R*_P_ by increasing the pH level. It should be noted that the linear plot beyond semicircle region stems from the Warburg impedance omitted in the equivalent circuit, which is not crucial parameters in this work. Figure 3D shows the mean values of *C*_p_ and *R*_P_ as a function of pH value. A decrease in the pH value from 8 to 4 resulted in an increase in the *C*_p_ from 1.80 to 2.04 nF/cm^2^ while the *R*_P_ decreased from 0.145 to 0.046 MΩ. This effect is driven by a higher concentration of cations in the electrolyte, which enhances *C*_p_​ by promoting cation adsorption and establishing a net charge across the IGZO surface. The decrease in *R*_p_​ and increase in *C*_p_​ simultaneously at lower pH suggests more efficient charge transfer dynamics at the IGZO interface, which in turn amplifies the absolute value of *I*_D​_ in transfer characteristics. Notably, these devices demonstrate a robust linear relationship between *R*_p_​ (R^2^ = 0.956), *C*_p_​ (R^2^ = 0.992), and pH levels across the pH range from 8 to 4, underscoring a predictable and precise response profile that could have significant implications for sensor applications.

Various physicochemical models have been suggested to elucidate the function of pH sensitivity at electrochemical interface. The most extensively accepted model employs the site-binding theory that was initially formulated to explain the surface charging in various electrolytes^23^. Figure 3E illustrates the suggested model for pH-dependent chemistry at the IGZO-electrolyte interface, and consequent redox behavior of IGZO, wherein the electric field is solely dependent on *V*_TG_. The neutral hydroxyl sites on the IGZO surface, which stems from surface dangling bond, can either donate or receive protons from the surrounding electrolyte, and the direction of interaction is affected by the pH of the electrolyte. The pH-dependent surface chemistry can be described as follows:

4 $${\rm IGZO{-}OH} \leftrightarrow {\rm IGZO{-}O}^{-} + {\rm H}^{+} \quad ({\rm pH} > 7)$$

5 $${\rm IGZO{-}OH} + {\rm H}^{+} \leftrightarrow {\rm IGZO{-}OH}_{2}^{+} \quad ({\rm pH} < 7)$$

Due to the electronegativity difference between protonated and deprotonated hydroxide, the surface potential is leveraged by pH, leading to the pH-dependent *V*_TH_ shift. On the other hand, the charge gradient upon pH alteration becomes more pronounced when the *V*_BG_ is additionally applied. Figure 3F illustrates a schematic model of the dual-bias-derived charge accumulation at the IGZO-electrolyte interface under acidic electrolyte conditions. Because the accumulated charge concentration, which determines surface potential, is governed by both pH condition and *V*_BG_ as discussed earlier, we speculate that this synergetic effect allows for more negatively shifted *V*_TH_ under identical pH variation, i.e. enhanced sensitivity.

### Sensitivity, stability, and potential of pH sensors based on DB EGTFT

**Fig. 4:**
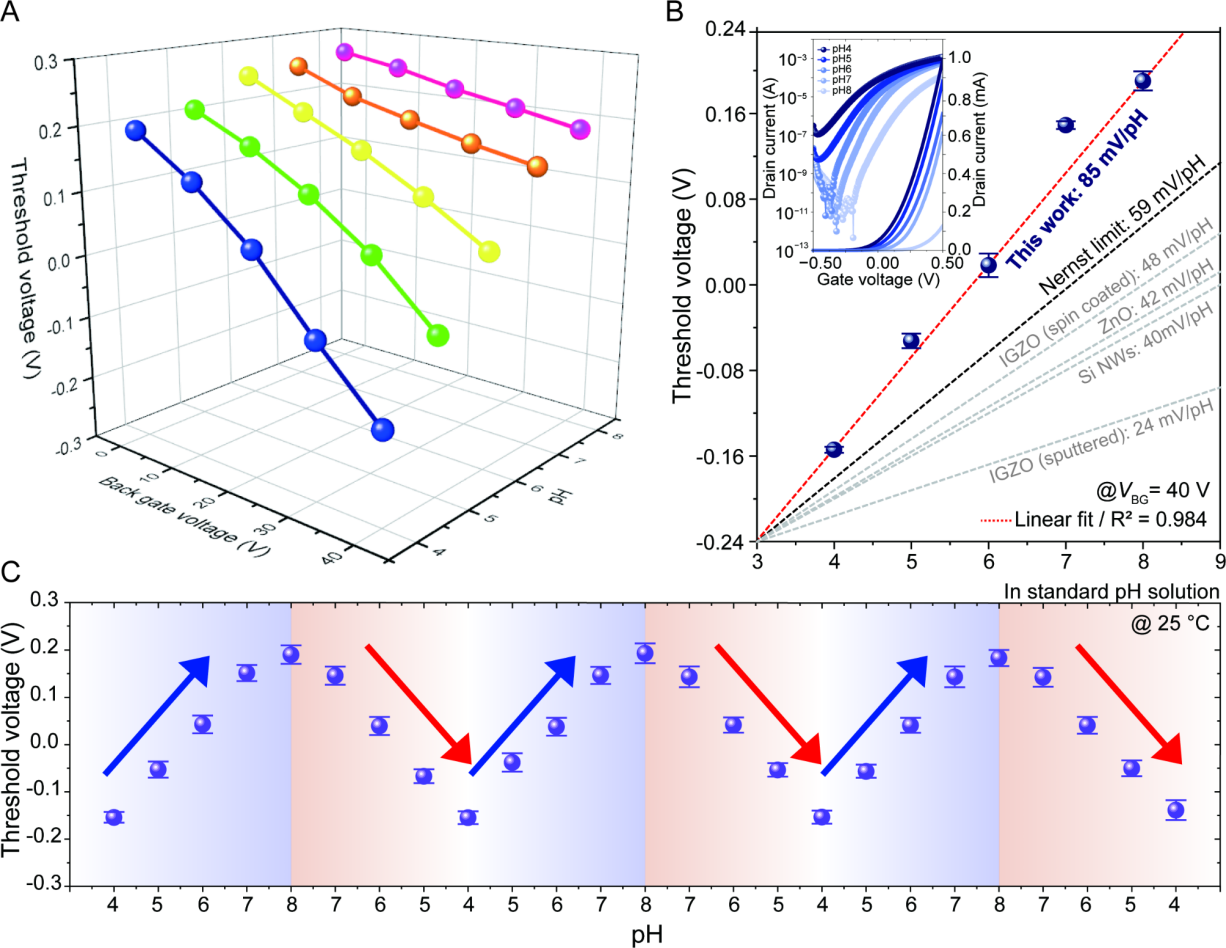
Electric characteristics of DB EGTFTs in various pH solutions at leveraged*V*_BG_ and pH levels. (**A**) Three-dimensional plots of pH and *V*_BG_ against *V*_TH_. (**B**) The plots of *V*_TH_ as a function of pH with the pH sensitivity, encompassing a comparison of slopes with the Nernst limit and various other materials. The inset shows the transfer characteristics with various pH solutions at *V*_BG_ = 40 V. (**C**) The *V*_TH_ dynamics of dual-gate pH sensors under repetitive pH cycles in pH standard solutions, where *V*_D_ = 0.5 V, *V*_TG_ = − 0.5–0.5 V, and *V*_BG_ = 40 V.

As demonstrated earlier, the pH sensitivity depends on both *V*_BG_ and pH levels. Figure S5 showcases the transfer curves of DB EGTFTs, where *V*_BG_ and pH levels were varied ​​from 4 to 8, and *V*_BG_ from 0 to 40 V, respectively. Figure 4A presents the extracted *V*_TH_ at each condition, exhibiting consistency with previous results. It can be seen that the Δ*V*_TH_ was directly affected by a change in the interfacial capacitance with pH variation under the fixed *V*_BG_. Figure 4B shows the plots of *V*_TH_ as a function of pH at *V*_BG_ = 40 V. From the calculations, we achieved an optimal sensitivity of 85 mV/pH with a high degree of linearity, surpassing those of previously reported EGTFTs using diverse sensing membranes, including sputtered IGZO, RuO_2_-SnO_2_, silicon (Si) nanowires, and ZnO (Table S1)^18,40–43^. Note that, unlike other extended gate-based IGZO pH sensors that operate within voltage windows above 5 V to achieve sensitivities beyond the Nernst limit^30–34^, we defined a boundary condition in which the active layer itself functions as the sensing layer, enabling us to achieve high sensitivity within a narrow sub-0.5 V operating range.

Finally, we verified the operational stability of the pH sensor for practical applications. A cyclic pH variation can induce non-ideal phenomena such as hysteresis effect, which is typically attributed to interfacial interactions or slow ionic transportation in the electrolyte. However, the proposed devices substantiated reliable operation, indicating chemical durability under acidic and basic electrolyte, as well as reduced interfacial traps. Figure 4C shows the *V*_TH_ plots of DB EGTFTs under a pH cycle loop of 4 → 8 → 4 → 8 → 4→ 8→ 4. For statistical investigations, we measured 15 devices in the same manner as for the pH cycle (Figure S6).

### pH sensors based on DB EGTFT in PBS solution and artificial urine

**Fig. 5:**
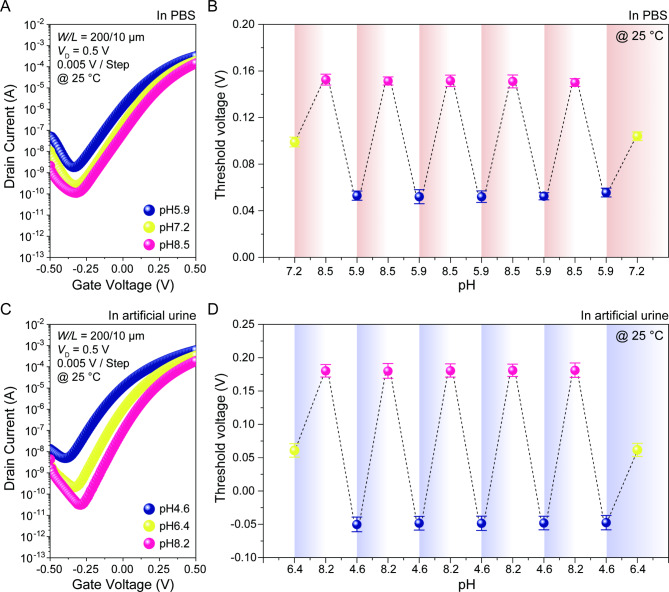
Electric characteristics of DB EGTFTs in various biofluids. Transfer curve of dual-gate pH sensor in response to various pH levels for (**A**) PBS solutions (at 5.9, 7.2 and 8.5) and (**C**) artificial urine (at 4.6, 6.4 and 8.2). The *V*_TH_ dynamics of dual-gate pH sensors under repetitive pH cycles in (**B**) PBS solutions and (**D**) artificial urine, where *V*_D_ = 0.5 V, *V*_TG_ = − 0.5–0.5 V, and *V*_BG_ = 40 V.

The sensitivity of pH sensors, even in the presence of various cations, anions and organic substances such as creatinine and urea, was thoroughly investigated for practical applications. Figure 5A and C show the pH-dependent transfer characteristics of the DB EGTFTs in PBS solution and artificial urine, where the pH levels of each solution were carefully titrated using acidic and basic chemicals. Coupled with the transfer characteristics, the DB EGTFTs exhibited reliable cyclic operations in each solution (Figs. 5B, D). The devices demonstrated the pH sensitivity beyond the Nernst limit, reaching up to 63 mV/pH in both artificial biofluids (Table S2), though the values were slightly lower than the standard pH solutions. Diverse ion species in complex media, particularly alkali metal ions and/or halides, were drawn towards the IGZO surface by electrostatic forces, which interfered with the pH-dependent protonation and deprotonation processes. Thses were regarded as the predominant reasons for reduced pH sensitivity compared to that in standard solutions. Nevertheless, the diverse ion species in artificial biofluids, even at relatively high concentrations, did not alter the pH level (Table S3). This ensured the operational reliability of the DB EGTFTs, paving the way for high-performance pH sensors for practical point-of-care diagnostic applications.

## Conclusions

We developed a highly sensitive pH sensor based on DB EGTFTs without additional surface modification of the IGZO sensing layer. The sol-gel methodology facilitated uniform deposition of the IGZO film across a 6-inch wafer containing 1200 individual EGTFTs, enabling long-term stability up to 1000 s, albeit in single-gate operation mode. Verifying the *V*_BG_-dependent drift of *V*_TH_, we demonstrated that the proposed pH sensors based on DB EGTFT yielded not only the enhanced sensitivity of up to 85 mV/pH, but also reliable *V*_TH_ fluctuations under pH changes from 4 to 8. Furthermore, the proposed pH sensors demonstrated stable and consistent pH sensing in artificial urine and PBS solutions, where the pH levels of the physiological electrolytes were alternately adjusted to acidic and basic conditions. In-depth electrochemical analyses elucidated the role of *V*_BG_ in supplying additional electrons in the IGZO channel, thereby expanding *V*_TH_ windows. Our cost-effective, easy-to-fabricate, and high-performance sensing platform presents a compelling option for developing innovative, affordable, and highly sensitive devices suitable for water quality monitoring and point-of-care diagnostic applications.

## Electronic supplementary material

Below is the link to the electronic supplementary material.

Supplementary Material 1

## Data Availability

The datasets generated during and/or analyzed during the current study are available from the corresponding author on reasonable request.
