# Supplementary material for: Dual-biased metal oxide electrolyte-gated thin-film transistors for enhanced protonation in complex biofluids
Source: Sci Rep. 2024 Dec 28;14:30772. doi: 10.1038/s41598-024-80005-0 (PMC11680892; doi:10.1038/s41598-024-80005-0)
Supplement: Supplementary file 1 — Supplementary Material 1 [file 41598_2024_80005_MOESM1_ESM.docx]

**Dual-biased metal oxide electrolyte-gated thin-film transistors for enhanced protonation in complex biofluids**

*Chuljin Hwang*^a^, *Yoonseok Song*^b^, *Seokhyeon Baek*^b^, *Jun-Gyu Choi*^a^*, and *Sungjun Park*^a,b^*

*^a^Department of Electrical and Computer Engineering, Ajou University, Suwon 16499, Republic of Korea*

*^b^Department of Intelligence Semiconductor Engineering, Ajou University, Suwon 16499, Republic of Korea*

*Corresponding Authors: Dr. Jun-Gyu Choi (jungyuchoi93@ajou.ac.kr), Prof. Sungjun Park (sj0223park@ajou.ac.kr)


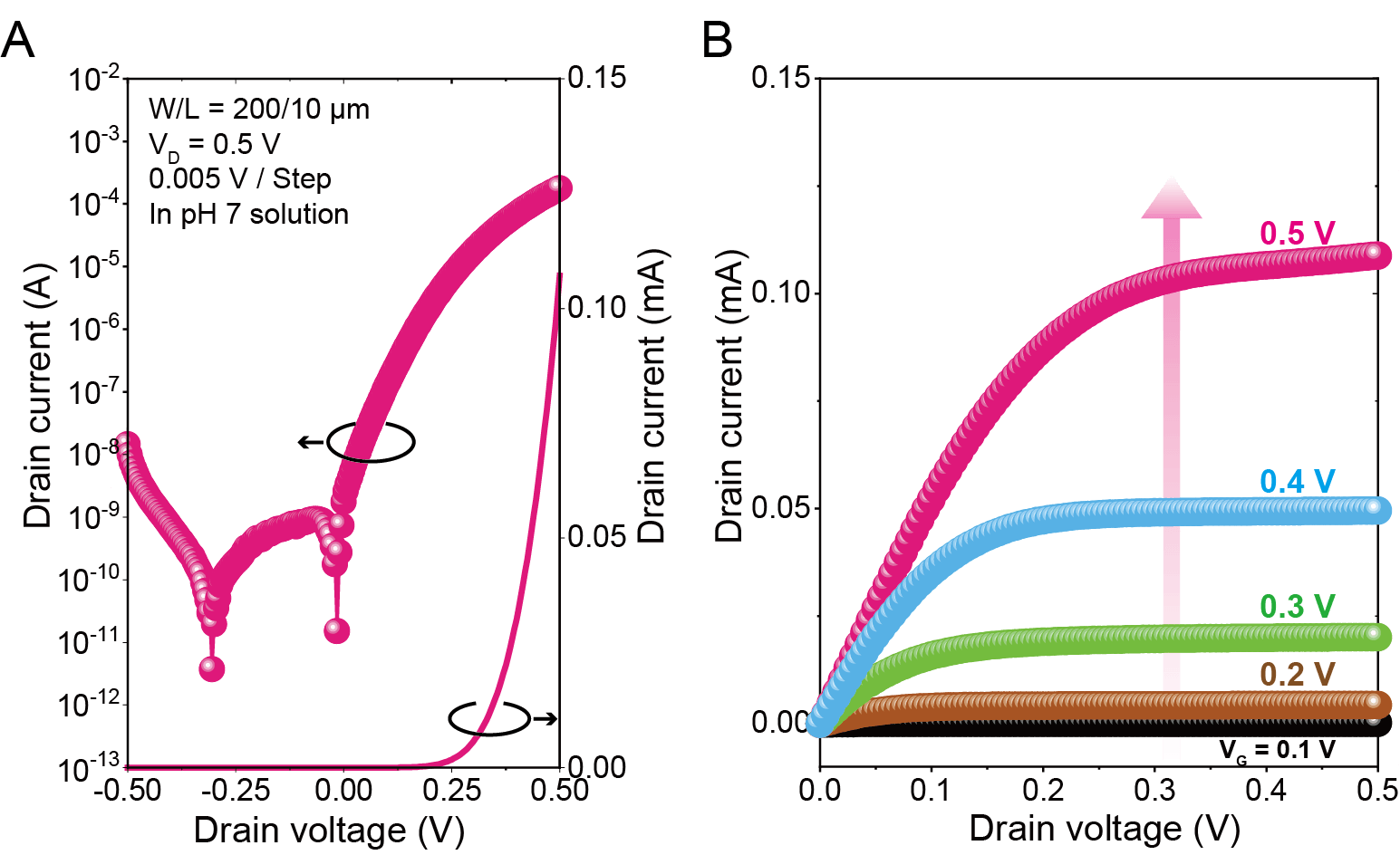


**Supplementary Figure 1.** (A) Transfer characteristics (*V*_G_ vs *I*_D_) measured at *V*_DS_ = 0.5 V (*V*_GS_ = −0.5–0.5 V) and (B) output characteristics (*V*_DS_ vs *I*_DS_) measured at *V*_GS_ = 0.1, 0.2, 0.3, 0.4, and 0.5 V in pH 7 solution. The IGZO EGTFTs have channel width and length of 200 and 10 μm, respectively.

**
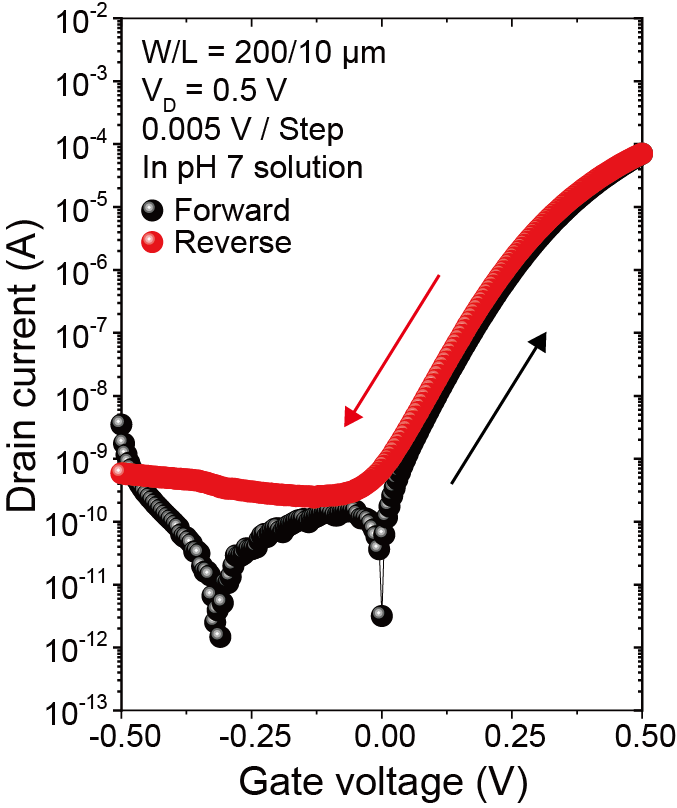
**

**Supplementary Figure 2.** Hysteresis curve of the IGZO EGTFTs with a step of *V*_G_ = 0.05 V at *V*_D_ = 0.5 V at room temperature in pH 7 solution. The IGZO EGTFTs have channel width and length of 200 and 10 μm, respectively.

**
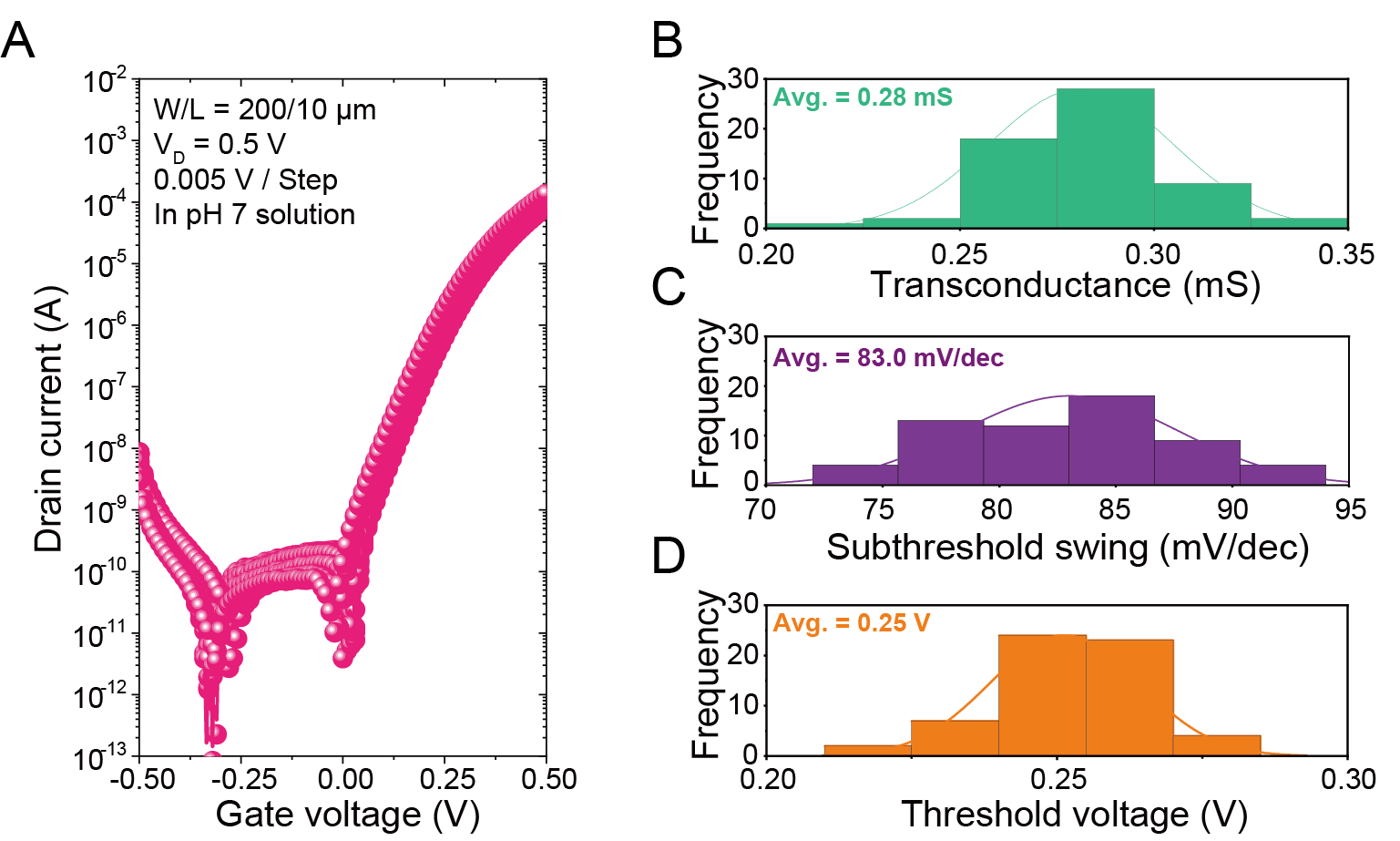
**

**Supplementary Figure 3.** (A) Representative transfer characteristics (*I*_DS_–*V*_GS_) of IGZO-EGTFTs (a total of 60 devices) in the 6-inch wafer were measured. All experiment results were applied with a fixed drain voltage of 0.5 V. The IGZO EGTFTs have channel width and length of 200 and 10 μm, respectively. Histogram of (B) transconductance, (C) subthreshold swing and (D) threshold voltage from a total of 60 devices.

t


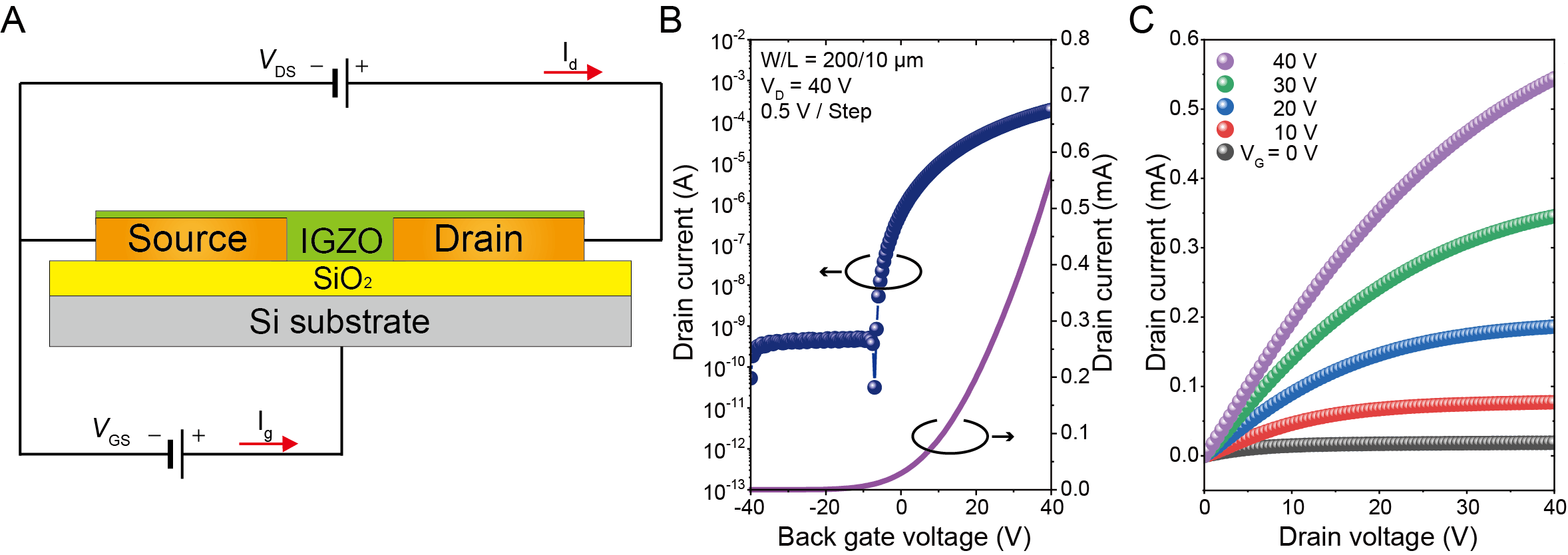


**Supplementary Figure 4.** (A) Schematic diagram of the bottom gate structure and electrical connections of an IGZO TFTs (B) Transfer (*I*_D_-*V*_G_) and (C) output characteristics (*I*_D_-*V*_D_) under pH 7 solution. The IGZO EGTFTs have channel width and length of 200 and 10 μm, respectively.


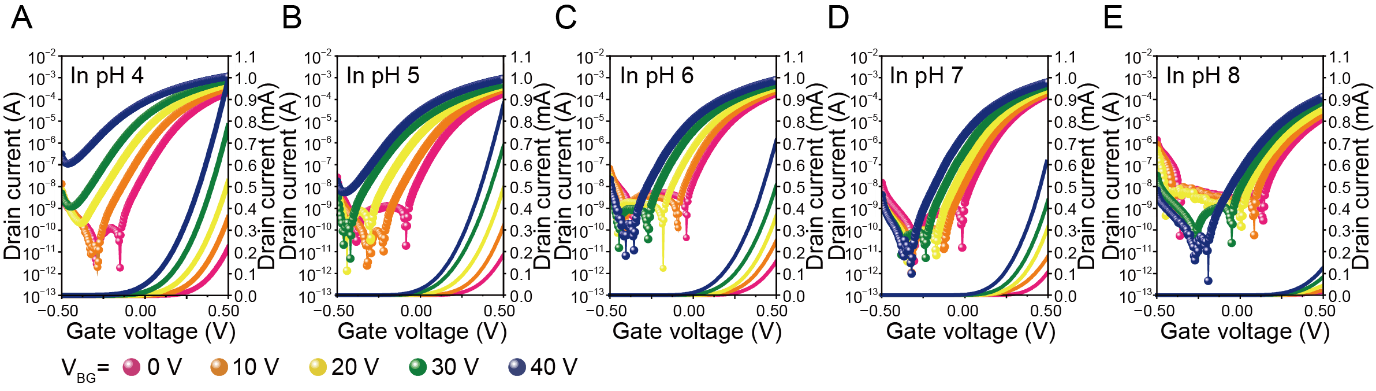


**Supplementary Figure 5**. Electric characteristics of dual-gate mode with varying bottom gate voltage and pH solution ((A)–(E): pH 4, 5, 6 7, and 8, respectively). The IGZO EGTFTs have channel width and length of 200 and 10 μm, respectively.


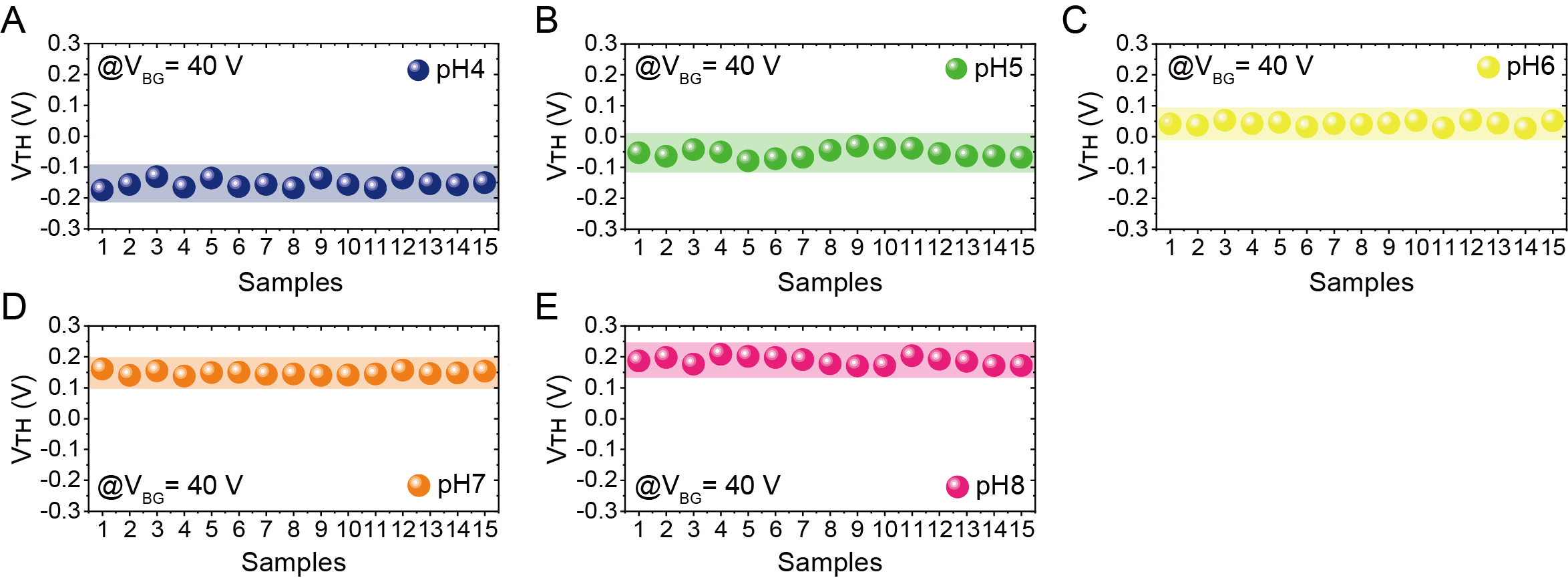


**Supplementary Figure 6.** Plot describing the sensing characteristics according to pH levels from 4 to 8 at *V*_BG_ = 40 V ((A)–(E): pH 4, 5, 6 7, and 8, respectively).

**Supplementary Table 1.** Comparison of the parameters of pH sensors

| **Materials** | **Deposition**  **technique** | **Structure** | **Operation voltage (V)** | **Electrical**  **Technique** | **Linear range (pH)** | **Sensitivity (mV/pH)** | **Samples** | **Ref.** |
| --- | --- | --- | --- | --- | --- | --- | --- | --- |
| ZnO | Vapor cooling condensation | Single gate | 0 – 12 | I-V | 4-12 | 42 | pH solution | [1] |
| RuO_2_–SnO_2_ | Screen printing | Single gate | - | EIS | 2-12 | 56.5 | Tap water,  River water,  Lemon juice | [2] |
| IGZO | Sputtering | Single gate | -2 – 2 | I-V | 4-9 | 24 | pH solution | [3] |
| IGZO | Sputtering | Dual gate | -2 – 5 | I-V | 3-10 | 129.1 | pH solution | [4] |
| IGZO | Sputtering | Dual gate | -5 – 5 | I-V | 4,7,9 | 160 | - | [5] |
| IGZO | Spin coating | Dual gate | -0.5 – 0.5 | I-V | 4-8 | 85 | Artificial urine, PBS | This study |

**Supplementary Table 2.** pH sensitivity in PBS solution and artificial urine. The mean and standard deviations measured from a total of 22 devices.

**
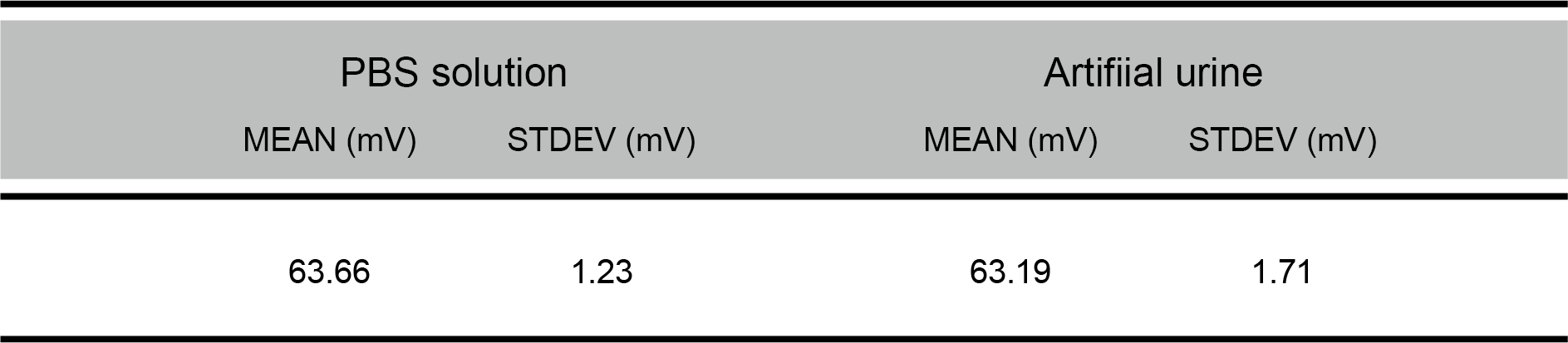
**

**Supplementary Table 3.** Chemical components of artificial urine and PBS solution


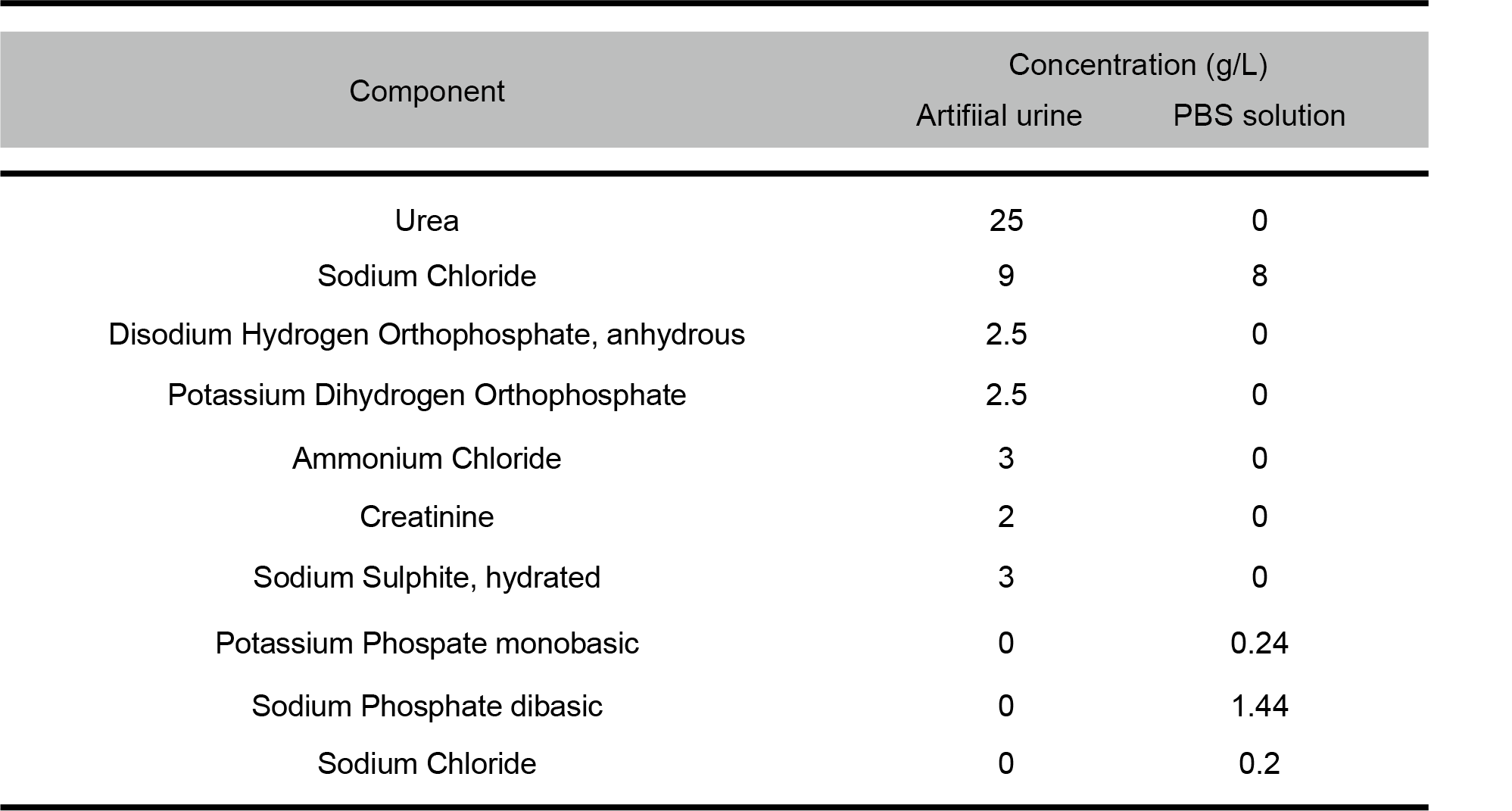


**References**

1. Lee, C.-T.; Chiu, Y.-S.; Lou, L.-R.; Ho, S.-C.; Chuang, C.-T. Integrated pH Sensors and Performance Improvement Mechanism of ZnO-Based Ion-Sensitive Field-Effect Transistors. *IEEE Sens. J.* **2014**, *14*, 490–496, doi:10.1109/JSEN.2013.2285488.

2. Manjakkal, L.; Cvejin, K.; Kulawik, J.; Zaraska, K.; Szwagierczak, D.; Stojanovic, G. Sensing Mechanism of RuO2–SnO2 Thick Film pH Sensors Studied by Potentiometric Method and Electrochemical Impedance Spectroscopy. *J. Electroanal. Chem.* **2015**, *759*, 82–90, doi:10.1016/j.jelechem.2015.10.036.

3. Kumar, N.; Kumar, J.; Panda, S. Enhanced pH Sensitivity over the Nernst Limit of Electrolyte Gated A-IGZO Thin Film Transistor Using Branched Polyethylenimine. *RSC Adv.* **2016**, *6*, 10810–10815, doi:10.1039/C5RA26409J.

4. Jang, H.-J.; Gu, J.-G.; Cho, W.-J. Sensitivity Enhancement of Amorphous InGaZnO Thin Film Transistor Based Extended Gate Field-Effect Transistors with Dual-Gate Operation. *Sens. Actuators B Chem.* **2013**, *181*, 880–884, doi:10.1016/j.snb.2013.02.056.

5. Kumar, N.; Kumar, J.; Panda, S. Back-Channel Electrolyte-Gated a-IGZO Dual-Gate Thin-Film Transistor for Enhancement of pH Sensitivity Over Nernst Limit. *IEEE Electron Device Lett.* **2016**, *37*, 500–503, doi:10.1109/LED.2016.2536359.
